# Epigenetic inheritance systems contribute to the evolution of a germline

**DOI:** 10.1098/rstb.2015.0445

**Published:** 2016-08-19

**Authors:** Michael Lachmann, Eric Libby

**Affiliations:** Santa Fe Institute, Santa Fe, NM 87501, USA

**Keywords:** differentiation, soma, hypercycle, graph, specialization

## Abstract

Differentiation within multicellular organisms is controlled by epigenetic markers transmitted across cell division. The process of differentiation will modify these epigenetic markers so that information that one cell type possesses can be lost in the transition to another. Many of the systems that encode these markers also exist in unicellular organisms but do not control differentiation. Thus, during the evolution of multicellularity, epigenetic inheritance systems were probably exapted for their current use in differentiation. We show that the simultaneous use of an information carrier for differentiation and transmission across generations can lead to the evolution of cell types that do not directly contribute to the progeny of the organism and ergo a germ–soma distinction. This shows that an intrinsic instability during a transition from unicellularity to multicellularity may contribute to widespread evolution of a germline and its maintenance, a phenomenon also relevant to the evolution of eusociality. The difference in epigenetic information contents between different cell lines in a multicellular organism is also relevant for the full-success cloning of higher animals, as well as for the maintenance of single germlines over evolutionary timescales.

This article is part of the themed issue ‘The major synthetic evolutionary transitions’.

## 1. Introduction

The genotype of a multicellular organism is the information that was passed down along the lineage from its ancestors. All cells in the organism share this genotype. A developmental programme is then used to reliably reproduce a differentiated multicellular body with its multiple cell types from a single genotype [1,2]. These genetically encoded programmes govern not only when cells differentiate but also which cell phenotypes are produced. Waddington [3] coined the term ‘epigenotype’ to describe the sum total of the patterns of development that a particular genotype manifests during the process that leads from a fertilized egg to an adult phenotype (see also [4]). Physically, most of the genotype is manifest in the DNA of the organism. The epigenotype, the current phenotypic state of the cells, is manifest in the expression levels of mRNAs and proteins, their current structure and location in the cell, etc. Epigenetic information can also be passed in cell division [5–7], and sometimes also across generations [8].

Because cellular state is determined by epigenetic information and organisms can and must change these states during differentiation, it follows that epigenetic information is changed and lost during development. This relative instability of epigenetic information may have important consequences for primitive forms of multicellularity. Indeed, in this paper, we show that it can lead to the evolution of reproductive division of labour via a dedicated germline.

One of the best-understood epigenetic inheritance systems (EIS) is the methylation marking system, where the presence of methyl (CH_3_) groups on some cytosines (in most vertebrates and plants these are cytosines that have guanines as neighbours) or other nucleotides is transmitted from one cell generation to the next [9]. Inactive genes are often highly methylated, whereas the same genes may be transcribed if the methylation level is low. Developmental and environmental cues lead to changes in methylation, so the same gene may carry distinctly different methylation patterns (marks) in different cell types [10]. In addition to methylation marks, there exist other types of marks, involving DNA-associated proteins that affect gene activity and can also be transmitted in cell lineages, and are maintained and reconstituted following DNA replication [11]: histone markings are also thought to be transmitted across cell division [5]. Differences in cell states can also be transmitted through positive regulatory feedback loops and other mechanisms (a more thorough review of these and other EIS is given in [9,12]).

Interestingly, EIS also exist in unicellular organisms. For example, bacteria and yeast cells have EIS and can transmit induced and accidental functional and structural states to their progeny [13,14]. Unicellular organisms do not, however, undergo epigenesis in the classical sense: this notion usually refers to processes of development and cell differentiation in multicellular organisms. Instead, unicellular organisms seem to use EIS to transmit cellular state information across generations, thereby acting as another, more malleable, inheritance system, or as an immune system acting in concert with restriction enzymes [15,16]. Thus, during the evolution of multicellularity, EIS were probably exapted for their current use in differentiation.

In the space of the multitude of independent evolutionary origins of multicellularity, and many hundreds of millions of years of independent evolution of multicellular species, many different reproductive and developmental abilities in cells evolved: totipotent, pluripotent and multipotent stem cells, and various gradations within these. There are also many uses of the words germline and soma. In this paper, we follow the definition used by Woodland [17] for primordial stem cells: any cell that usually forms either germ cells or soma. Soma will be any cell that usually cannot form germ cells. For simplicity, we model only asexual reproduction; any cell type that usually forms spores or differentiates into soma is germ, and any cell that usually cannot form spores is soma.

**Figure 1.**
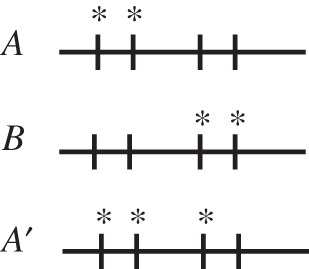
A hypothetical methylation pattern illustrates the loss of epigenetic information. Cell types *A* and *B* have different methylation patterns. Genetically encoded mechanisms transform *A* cell types to *B* and vice versa, during epigenesis. *A*’ is a new epimutant similar to *A* in the function that it performs in the multicellular body. The process that transforms *A* to *B* will also transform *A*’ to *B*. Since *B* can then not recreate *A*’, the epimutation is lost in differentiation.

During multicellular differentiation, epigenetic information is modified by genetically encoded mechanisms [1]. As a consequence, when a cell switches its phenotype, the differentiation mechanism transforms the epigenetic information of the previous phenotype into that of the next phenotype. If a cell acquires a chance modification to its epigenetic information, i.e. an epimutation that provides a fitness benefit to the organism, then this modification may be lost the next time the cell switches phenotypes (see figure 1 for an example). If lost, the epimutation will not be reliably produced again. There are two ways, however, in which this epimutation could be reliably produced. First, a genetically encoded mechanism could randomly gain a mutation enabling it to produce this epimutant. Second, a certain lineage could be sequestered such that it does not switch phenotypes, thereby establishing a germline.

Here we show that the potential loss of beneficial epimutations may drive the evolution of a germ–soma division, particularly in primitive forms of multicellularity. Because of the presence of EIS, a beneficial epimutation may be reliably copied to the offspring. Individuals carrying these epimutations compete with individuals that do not carry them—offspring of the same organism that derive from cell types in which this epigenetic marking is lost. Genetic mutations can then invade the population in which cells that lost the original epimutation forego their ability to produce a new organism. Using a simple mathematical model, we show in this paper that this occurs readily.

The argument in this paper can be summarized to the following: since differentiation is controlled by responding to and changing epigenetic states, it follows that some epigenetic information is erased during the process of development. If epigenetic information can also be transferred across generations, some cell types can accumulate beneficial epigenetic mutations that are lost during development as they differentiate into other cell types. The consequence is that when not all cells have the same epigenetic information, lineages of the multicellular organism produced by these cell types have a lower fitness than those produced by others. This difference in fitness can then lead to the evolution of a germline, where only cells that have the highest fitness lineages actually produce offspring for the organism.

## 2. Description of the model

To see how information loss can drive the evolution of reproductive division of labour, we model a primitive multicellular organism that is composed of two cell types, *A* and *B*, each of which can produce the other. The state of each cell is determined by epigenetic information that can either be passed on intact or modified by a genetic mechanism—the developmental programme. For clarity of communication, we will assume that the epigenetic information is encoded by DNA methylation, though the model could also apply to other EIS.

Each multicellular organism begins its life as a single cell, called a spore. The organism undergoes growth through cell division and differentiation. For simplicity, we assume that the developmental programme ensures that each fully developed adult organism expresses the same organization of cells. Thus, adults contain exactly *N_A_* cells of type *A* and *N_B_* cells of type *B*. We observe similar results from our model if we reduce the severity of this assumption such that multicellular organisms with other numbers of *A* and *B* cells are viable but less fit. However, this assumption simplifies the model by allowing us to avoid specifying fitness as a function of the number of *A* and *B* cells at reproduction.

**Figure 2.**
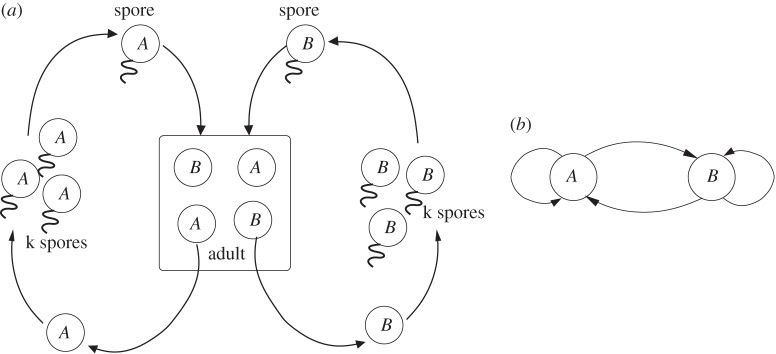
(*a*) The original life cycle of the organism. There is an adult stage with a fixed number of cells of two types, *A* and *B*. Both cell types can produce spores that maintain their epigenetic identity, and can reproduce/differentiate into a new adult. (*b*) Differentiation graph of the organism. Nodes represent cells of an epigenetic type, arrows represent the possibility for a cell type in the spore to produce another cell type in the adult through replication or differentiation.

As stated above, this model describes an organism without a dedicated germline. This means that the initial cell of the organism is not necessarily of a certain epigenotype—it could be of type *A* or of type *B*. In our model, the first cell, regardless of type, reproduces and differentiates to produce the fully developed adult organism composed of *N_A_ A* cells and *N_B_ B* cells. We assume that this takes place by the cell first reproducing itself a number of times and then switching to produce the opposite cell type. Adult multicellular organisms reproduce asexually via spores—the implications of sexual reproduction, and in particular of diploid organisms, are addressed in the discussion. The number of spores produced by an adult is under genetic control, but initially we assume that each cell in the adult produces exactly *k* spores. Consequently, *A* cells produce *k* spores of type *A*, each of which contains the same epigenetic information and will constitute the first cell of type *A* in a new organism. Figure 2 shows the life cycle of these organisms and the differentiation graph.

**Figure 3.**
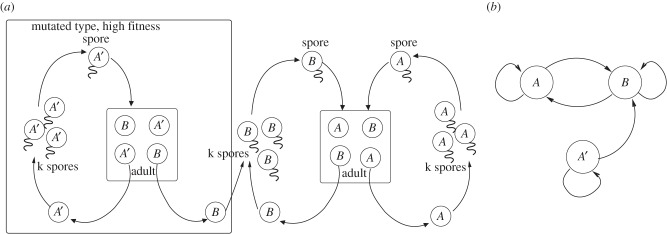
(*a*) Modified life cycle of the system with cells of type *A*, *B* and *A*′. (*b*) Differentiation graph with the epimutation *A*′. Notice that cells of type *A*′ produce cells of type *A*′ and *B*, but that cells of type *B* cannot produce cells of type *A*′.

We assume that at some point the epigenetic pattern of a cell of type *A* undergoes an epimutation and produces a cell of type *A*′. The change occurs in the epigenetic information, and not in the genetic sequence—its genes are still identical to the genes of all other cells in the organism (see figure 1 for possible methylation patterns of cells of type *A*, *B* and *A*′). Since the epimutation is part of an epigenetic inheritance system, cells of type *A*′ can reproduce the *A*′ state. This means that: (i) *A*′ cells produce spores of type *A*′ and (ii) organisms originating from such spores will contain *N_A_* cells of type *A*′. We further assume that the genetic developmental mechanisms that cause cells of type *A* to become type *B* will also cause cells of type *A*′ to become cells of type *B*. Thus, once development occurs and a *B* cell is produced, the information of whether the parent cell was *A* or *A*′ is lost. When spores of the *B* cell differentiate, they will give rise to *A* cells. Figure 3 shows the life cycle and differentiation graph with the new *A*′ epimutant.

We assume that the epimutation that gave rise to an *A*′ cell has fitness consequences. Multicellular organisms with cells of type *A*′ and *B* have significantly higher fitness than organisms with cells of type *A* and *B*. Adult organisms with higher fitness produce more spores: in organisms with *A*′ cells, both *A*′ and *B* cells produce *k* · *f*′ spores each, where *f*′ > 1 represents the fitness advantage.

## 3. Formal description of the model

Denote the frequencies of spores of epigenetic type *i* at the beginning of a generation as *p_i_*. The three epigenetic types are *A*, *B* and *A*′ (in the following ‘type’ refers to epigenetic type). The number of cells of type *j* in an adult that started from a spore of type *i*, denoted by *G_ij_*, is
3.1
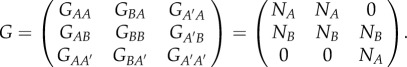
This matrix represents the differentiation graph of the system, in which an entry at position *i*,*j* represents the number of cells of type *j* that are produced in an adult that originates from a spore of type *i*.

The fitness of an adult that started as a spore of type *i* is denoted as *f_i_*. Notice that in this paper, we track fitness of organisms by the number of spores that are produced and survive from generation to generation. This fitness can be represented by the following diagonal matrix:
3.2
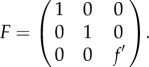
The number of cells of the various epigenetic types in the population in the adult stage is *F* × *G* × ***p***, and the number of spores of the different types in the next generation is *k* · *F* × *G* × ***p***. The total number of spores produced is ***1*** × *k* · *F* × *G* × ***p***, where we denote ***1*** ≡ (1, 1, 1). Thus, the distribution of spores in the next generation, is
3.3
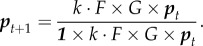


The equilibrium distribution of this system depends on the eigenvalues of the matrix *F* × *G*, which is
3.4
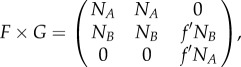
when *f*′*N_A_* is bigger than the largest eigenvalue of the upper left-hand 2 × 2 matrix
3.5
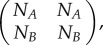
i.e. bigger than *N_A_* + *N_B_*, then the eigenvector with the largest eigenvalue of the system cannot be of the form (*a*, *b*, 0)—it must have a non-zero third component—and thus *A*′ is present in the equilibrium population. So if
3.6
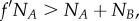
then *A*′ will successfully invade the population. In that case, the growth rate of the whole population, the largest eigenvalue of the matrix, is *f*′*N_A_*, which is the growth of type *A*′. Therefore, type *A*′ acts as a source for the population, and types *A* and *B*, with lower growth rates, act as sinks.

## 4. Reproductive division of labour

So far, we have assumed that every cell in the adult produces the same number of spores (*k* or *k* · *f*′). We now add an additional assumption that makes it possible for a cell to specialize in reproduction. We assume that the genome determines allocation of resources in a certain cell type, either to spore production or aiding spore production of other cells. This could correspond to a situation where cells preparing to become spores are packed with resources to maximize the number of spores they can produce. If one cell forgoes this process, more resources are available to other cells, thereby increasing the number of spores they produce.

For simplicity, we assume that resources are re-distributed among cells in an adult according to a parameter *r* which corresponds to each cell type's role in spore production, i.e. their reproductive ability. For example, cells of type *B* use a proportion *r* of their resources to produce spores and a proportion 1 − *r* is contributed to a general pool that is divided among all cells in the adult, in proportion to their reproductive ability. If all cells have the same value for *r* then all will produce the same number of spores. If *B* cells have *r* = 0 and *A* cells have *r* = 1 then only *A* cells will produce spores.

We allow for the possibility that transferring resources between cells incurs some loss. The proportion of resource lost, *L*, is called the loss factor—the amount of resources *R* are reduced to (*R* − *LR*). A negative value for *L* corresponds to a benefit from specialization: loss of reproductive ability of one cell type is more than compensated by the gain in another cell type. The total of *R* resources are then increased to (1 + |*L*|)*R*. In this paper, we concentrate on the opposite case, *L* > 0, though most of our claims also apply for *L* < 0.

The number of spores of type *i* produced in the next generation in an organism with fitness *f* is
4.1
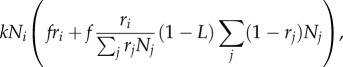
where *r_i_* is the reproductive ability of cells of type *i*. This is a linear model, in which the reproductive ability given up by some cells is divided among all cells in the organism in proportion to their reproductive ability. Summing over *i* in equation (4.1) the total number of spores produced by the organism is:
4.2
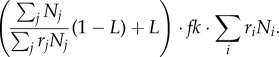
If we substitute 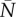 for 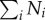 and 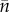 for 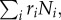
then the total number of spores produced by the organism is

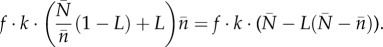
When the reproductive ability of all cells is 1, the total number of spores produced is 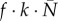 so that when the reproductive ability of some cells is smaller than 1, the organism gives up the production of 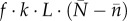 spores.

Before the invasion of the epimutation *A*′ and for 0 ≤ *L* < 1, any mutation that increases *r_i_* will invade. As a result, all cell types will reproduce equally at *r_A_* = *r_B_* = 1. After the invasion of the epimutation *A*′, any mutation that decreases *r_B_* will invade. As a result, cells of type *B* will give up their reproductive ability, down to *r_B_* = 0. These cells will become soma and cells of type *A*′ will become germ.

**Figure 4.**
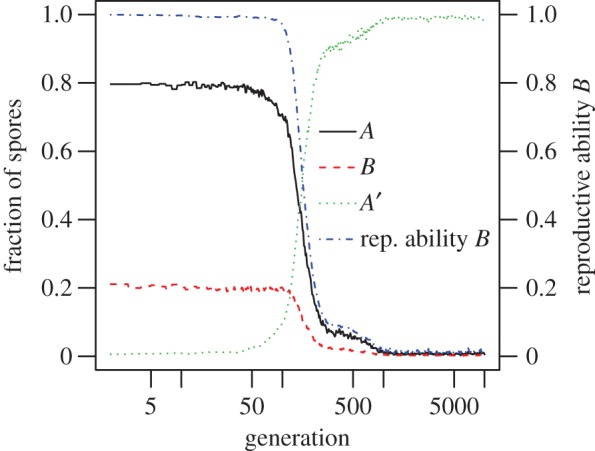
Evolution of a germline. At generation 1 epimutation *A*′ was introduced. A series of mutations caused the reproductive ability of *B* to decline to almost 0. The *A*′ is equivalent to a germline at the end of the run, and *B* soma. *N_A_* = 4, *N_B_* = 1. *f* = 1, *f*′ = 1.3, *k* = 4, *L* = 0.5 population size is 10^3^. Graphs show proportion of spores of the different types over time, and the average reproductive ability of *B* over time. Shown are results of one typical run.

The model as described in the previous section was simulated for a population size of 1000. Figure 4 shows the results from a typical run. At first, the reproductive ability of *B* stays at 1. Once *A*′ invades the population, the reproductive ability of *B* declines until it reaches almost 0 by generation 1000.

One can ask what happens if through genetic changes cells of type *B* gain the ability to produce cells of type *A*′ instead of *A*. Such a mutation will invade the population. In that case, the life cycle of the organism will return to the one described in figure 2. The fitness benefit provided by such a mutation declines as the reproductive ability of *B* declines. When *B* loses all reproductive ability, i.e. is purely soma, there is no selection pressure for the ability of *B* to produce cells of type *A*′. However, if and when a genetic mutation causes *B* to gain the ability to produce *A*′, selection will favour regaining the reproductive ability of *B*. In this case, the bigger the loss factor *L*, the faster *B* will regain its reproductive ability. When *L* = 0, there is no pressure for *B* to regain its reproductive ability. So with a big loss factor, *B* will lose its reproductive ability slowly and regain it quickly; with a small loss factor, *B* will lose its reproductive ability quickly and regain it slowly.

## 5. Other differentiation graphs

Until now we considered a particular differentiation graph with two states. In this section, we show that our results are general and hold for differentiation graphs with any number of states.

Why should one consider cases with more than two cell types? We have shown that two phenotypic states present enough opportunity to permit evolution of a germ–soma division of labour. Furthermore, Simpson [18] has pointed out that the germ–soma division is often an early event in the evolution of multicellularity and sociality. It would then seem that the case with more than two phenotypic states, without a dedicated germline would be hard to evolve in the first place. Yet, it is possible that even in a case with a dedicated germline, a genetic mutation could enable a somatic cell type to be a germline as well. This would lead to a case of *n* cell types with two germ cell lines. As we show in this section, these cases are also unstable and will ultimately result in one germline being lost in favour of the other.

**Figure 5.**
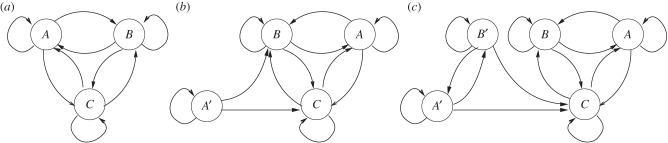
Differentiation graph for three cell types. Nodes represent cell type, arrows indicate that the cell type pointed to will be present in an adult that originated in a spore of the originating cell type. (*a*) Original differentiation graph. (*b*) Differentiation graph with epimutation in which *A* mutates to *A*′, and this epimutation is lost in the differentiation to *B* and *C*. (*c*) Differentiation graph in which *A* is mutated to *A*′. The epimutation is retained in differentiation to *B*, which leads to the creation of *B*′. The epimutation is lost in differentiation of *A*′ and *B*′ into *C*.

To generalize our results, we assume that some cell types will undergo an epimutation while preserving the basic differentiation graph. As before, the epimutation will be lost along some differentiation pathways. First, we will go through two examples of a differentiation graph of size 3. Figure 5*a* shows a differentiation graph with three nodes, and in (*b*,*c*) there are two possible epimutations that have the properties mentioned above. In figure 5*b*, *A* mutates into *A*′, but the epimutation is lost when *A*′ differentiates both into *B* and into *C*. In figure 5*c*, *A* mutates into *A*′, and in the differentiation process that made *B* out of *A* the mutation is not lost, so that *A*′ becomes *B*′. The mutation is lost, instead, in the transition from *B* to *C* so that *B*′ becomes *C*.

As before, we use differentiation graphs in which each node is a cell type, and an arrow between nodes *A* and *B* means that a spore of type *A* will differentiate to make a cell of type *B* in the adult. We represent this graph with a matrix, in which each row/column corresponds to a cell type, and entry *i*,*j* indicates how many cells of type *j* in the adult are produced by a spore of type *i*. Let us consider an organism with *n* cell types, and with a differentiation matrix *M*. An epimutation occurs and produces potentially *n* + *n* cell types because each of the *n* types may possess the epimutation. At first, let us assume that the mutation is preserved through the whole differentiation processes. In that case the new differentiation graph can be represented by the following 2*n* × 2*n* matrix:

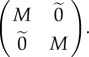
in which 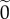 represents an *n* × *n* matrix with all entries 0. In this case, the epimutation acts like a genetic mutation since it is not lost or modified in differentiation. Thus, if an organism with the new cell type(s) has a higher fitness, then it will invade the population, just like a beneficial genetic mutation would. The eigenvector with the highest eigenvalue of the differentiation matrix times the fitness matrix will have either 0 in the first *n* entries, or 0 in the last *n* entries.

Now let us assume that somewhere in the differentiation process the epimutation is lost. In this case, the new differentiation matrix will take the form:

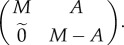
The matrix *A* represents the transitions in which the epimutation was lost. Since the epimutation cannot be regained in the differentiation process of the original organism, the lower-left corner of the new differentiation matrix remains 0—a cell without the epimutation cannot produce a cell in the adult that has it.

The eigenvector with the largest eigenvalue can take one of two forms: either the last *n* entries are all 0, in which case the epimutation did not invade, or there are non-zero entries among the last *n* entries in the eigenvector, in which case the epimutation is stable in the population. When the eigenvector is of the form 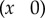, then *x* must be an eigenvector of the original differentiation matrix *M*. When the eigenvector is of the form 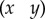, then *y* must be an eigenvector of the matrix (*M*−*A*). For the epimutation to be stable in the population, it must provide a fitness advantage that is bigger than the ratio of the largest eigenvalue of *M* to the largest eigenvalue of *M*−*A*.

Since the epimutation cannot be regained by the unmutated cells, and yet the epimutation is stable in the population, this means that as in the simple 2-cell case, the population with the epimutation acts as a source and the population without the epimutation acts as a sink for organisms with the epimutation. Therefore, as in the two-state case, there is a benefit to a genetic mutation that causes cells lacking the epimutation to give up their reproductive ability if they can contribute some of their resources to cells that still have it.

## 6. Continuous model

In the previous sections, we introduced a model with many simplifying assumptions to demonstrate the evolution of a germline. In this section, we analyse a slightly more realistic model of differentiation where some simplifying assumptions are removed.

**Figure 6.**
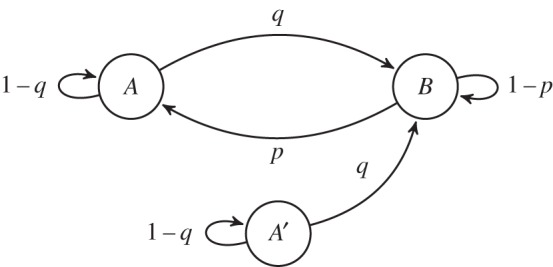
Differentiation graph for the continuous model. Arrows indicate transitions between epigenetic states and labels indicate the rates.

Similar to our previous models, we assume that a single genotype is able to epigenetically switch between two phenotypic states: *A* and *B*. We remove the restriction that the developmental programme tightly regulates how many *A* and *B* cells are produced. Rather, upon reproduction, a fraction of each phenotype switches to the other phenotype, *q* of *A* switches to *B* and *p* of *B* switches to *A* (see figure 6). As a consequence, adult multicellular organisms may have different numbers of *A* and *B* cells. Although adults may have different numbers of *A* and *B*, both are needed for the organism to be viable. We implement this dependence via sigmoidal functions of the form *f*(*X*) = *X*^2^/(*θ*^2^ + *X*^2^) in equation set (6.1), where *X* is the number of cells of a type, and *θ* is the threshold (here, *θ* = 10). Thus, *A* cells need at least some *B* cells in order to reproduce but once the number of *B* cells is above a threshold then there are diminishing returns to producing more *B* cells.

Again, we assume that a new epigenetic modification arises in the population called *A*′ which is similar to *A* in how it interacts with *B* and is lost when *B* switches its phenotype. In this model, the epimutant *A*′ does not directly affect the reproductive fitness of the multicellular organism, rather it simply reproduces at a faster rate than *A* (*s* > 0 in equation set (6.1)). We note that the growth of the population, i.e. d/d*t*[*A* + *A*′ + B], is equal to: *k_a_Af*(*B*) + (*k_a_* + *s*)*A*′*f*(*B*) + *k_b_Bf*(*A* + *A*′).
6.1
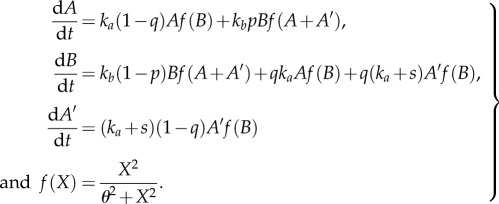
To model population growth, we numerically solve equation set (6.1) until the total population reaches a fixed number *N* (here, we choose *N* = 1000), which was *N* = *N_A_* + *N_B_* in the earlier models. Following growth to the carrying capacity, we apply a bottleneck that reduces the total population size to an initial size (2.01 in our simulations, where the very first instance is *A* = 1, *B* = 1, *A*′ = 0.01). The bottleneck reduces the population proportionally, e.g. the new value of *A* is 2.01(*A*(*t*))/1000, where *A*(*t*) is the number of *A* in a population of *N* = 1000. We then regrow the population back to carrying capacity and repeat the process until the initial conditions change less than some tolerance (Euclidean distance is less than 0.001).

**Figure 7.**
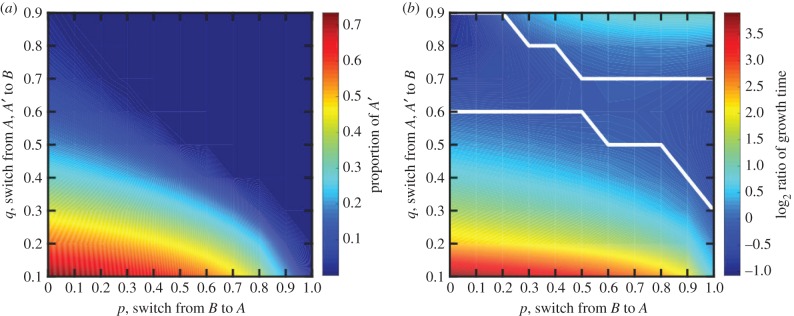
The effect of *A*′ in a continuous population. (*a*) The proportion of *A*′ in the stabilized population (after repeated growth and bottlenecks) is shown as a function of switch rates between types. Here, *A*′ has a fitness advantage of *s* = 1 over *A* types. The proportion of *A*′ is greater than 0 for all values tested though it is the highest when switching between phenotypes is infrequent. (*b*) The log_2_ ratio of times it takes populations to reach *N* when *B* types do not reproduce versus when they do is shown as a function of switching rates. The white line encloses the area in parameter space in which the population grows faster if *B* does not reproduce. This regime has low proportion of *A*′ in (*a*) and intermediate switch rates between types. Thus, when *B* cannot reproduce, the population can increase its production of *A*′ and grow faster.

We find that if *A*′ has a growth advantage over *A* then *A*′ increases in frequency. Figure 7*a* shows the proportion of *A*′ that stabilizes in the initial condition as a function of *p* and *q* for *s* = 1. The proportion of *A*′ is the highest for low values of *p* and *q* that correspond to infrequent switching between *B* and *A*. If *p* or *q* is too high, then the epimutation is lost.

By increasing in frequency, *A*′ can also reduce the time it takes the population (*A* + *A*′ + *B*) to grow to *N*. This is equivalent to increasing the speed of the developmental life cycle for the multicellular organism and thereby increasing its reproductive speed. For all combinations of *p* and *q* tested, we found that the presence of *A*′ increases the developmental speed of the organism. Indeed, for some combinations of *p* and *q*, organisms could reproduce faster if the reproductive ability of *B* were removed. Figure 7*b* shows the log_2_ ratio of population growth times, i.e. time until *A* + *A*′ + *B* = *N*, of the case where *B* reproduces (*k_b_* = *k_a_*) to the case where *B* does not reproduce (*k_b_* = 0). In area within the white lines, the population grows faster when *B* does not reproduce. This area corresponds to an area with a low proportion of *A*′ in figure 7*a*. Thus, by inhibiting the reproductive ability of *B*, the population increases the amount of *A*′ it produces and can grow faster as a whole.

## 7. Discussion

The phenomenon described in this paper is based on a simple premise: a multicellular lineage without a germ/soma distinction reaches an evolutionary impasse if certain cell types lose the ability to recreate the whole organism. There are two possible resolutions. First, the lineage can evolve some mechanism that restores the ability of all cell types to recreate the whole organism. Second, the lineage can evolve to transfer resources from the cell types that cannot recreate the whole organism to those that can. When the second path is taken, a germ/soma differentiation will occur.

**Figure 8.**
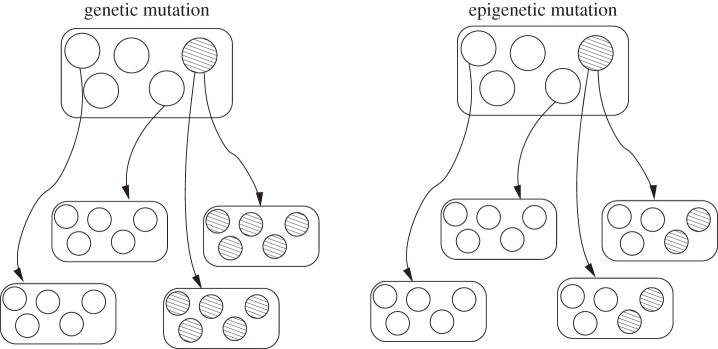
Comparison between inheritance of genetic and epigenetic mutations in an organism without germline. On the left, a genetic mutation occurs and is transmitted to all offspring that are produced by the mutated cell. On the right, an epimutation occurs. It is transmitted only to the offspring of the mutated cell and is lost in the differentiation process. In the offspring, however, both mutated and non-mutated cells exist.

To offer a scenario of how cells could lose the ability to recreate the whole organism, we used the distinct roles of epigenetic and genetic information in differentiation. In our case, a new epimutant arose that caused a ‘conflict’ between cells with and without the epimutation, which is then transmitted across generations. If instead, everything were under genetic control, the conflict only exists for a single generation (figure 8). The extended duration of the conflict in the epigenetic case provides an evolutionary opportunity for a resolution. Previous work [19,20] has pointed out that even a one-generation conflict, so long as it happens repeatedly, can be an evolutionary force to lessen the chance of mutations in the organism, or evolve a germ/soma division of labour.

Previous explanations for the evolution of germ/soma differentiation usually assume a benefit from specialization [21–26]. It is assumed that by having the soma specialize on non-reproductive tasks and germline on reproductive tasks, the organism as a whole gains more fitness than was lost through lack of reproduction of the soma. In our model, this would be equivalent to a negative value for *L*, which means that the loss of reproductive ability of one cell type is more than compensated by the gain in another cell type, leading to an overall increase in the number of offspring of the organism. While a negative *L* would lead to division of labour in our model as well, we chose to analyse the case of a positive *L*, corresponding to an overall loss of fitness when one cell type relinquishes some of its reproductive ability. The difference between this case and models of specialization is where the fitness benefits occur.

In traditional specialization models, the fitness gain is immediate. At the moment a soma is formed, the germ produce a larger number of viable offspring for the next generation. In our case, the germ produce a smaller number of viable offspring in the immediate generation. However, more viable offspring carrying the epimutation are produced, at the cost of producing less offspring not carrying the epimutation. Since the whole lineage originating from offspring that do not carry the epimutation has a lower fitness, it will ultimately be outcompeted by the lineage that does carry it. That means that far enough into the future, virtually all surviving lineages will be ones that carry the epimutation. Therefore, any increase in the number of offspring carrying the epimutation—even at the cost of a large decrease in those that do not—is beneficial. The benefit is not immediate but is realized in future generations. The condition for invasion of specialization thus is much weaker. Still, a first step was necessary: the epimutation had to provide a large enough fitness benefit to increase in the population. The process of invasion of germ/soma in our model therefore can be broken up into two phases: first, invasion of an epimutation through a strong fitness advantage, and then the invasion of specialization through a much smaller fitness benefit.

We model a scenario with asexual organisms, but multicellularity most often arose in sexual organisms [27,28]. In sexual organisms, recombination presents an additional constraint on the transmission of epigenetic information. If different cell types produce gametes with different epigenetic states, and the epigenetic information resides on the DNA, then recombinations of the different epigenetic markings have to be viable. This process could put strong pressure for uniformity of epigenetic information on the gametes. When the gametes are absolutely uniform with respect to epigenetic information, then the process outlined in the paper will not occur. If there is still some residual transfer of epigenetic information through the gametes, which is neutral with respect to recombination, then the process outlined in the paper could occur.

In some organisms, and in particular in plants, the germ/soma separation is not as strong as it is in higher animals—the organism can reproduce asexually from cells originating in many parts of the body. In those organisms, it might be possible to observe processes described in this paper. A possible empirical question could be: how fit are offspring that are produced by one cell type compared to those that are produced by another? Are there epigenetic mutations that are transmitted through one type, but erased during differentiation to another cell type?

What maintains the germline as the only cell type in the organism that can produce the next generation? One could imagine genetic mutations that allow production of germ from other cell types. Mostly, such mutations would be deleterious, but over millions of generations and environmental scenarios one can imagine that such a mutation could invade. In that case, we would have organisms where two or more cell types can produce germ. It could be that a mechanism such as the one described in this paper is responsible for continually selecting against a second germline—through accumulation of some beneficial epigenetic information in one of the germlines.

We discuss a model for the evolution of differentiation in multicellular organisms and show that reproduction through multiple germlines is not stable. The same process could also describe the evolution of a germline in social insects, such as wasps and termites. Here, the control of the state of an individual in the colony could be carried out by environmental interactions [29]. One would need to show that some of the epigenetic information of individuals in primitive colonies can be transmitted to the next generation of the colony. It has been shown that in Hymenoptera, germline/soma determination can be genetically controlled [30]. Here not all individuals in the colony are genetically identical. It would be interesting to test how such a genetic caste-determination system relates to our model.

The continuous model that we present does not need to be a multicellular organism in a strict sense; it simply requires that cells reach a carrying capacity quickly and transmit offspring to a new environment. This model could, therefore, apply to social interactions between genetically identical parasites within a host. It should be noted that such parasites have shown the ability to evolve a germ/soma division of labour as well (e.g. [31]).

Today, it is possible to artificially clone higher animals by using non-germline cells. When this is done, we are trying to artificially revert somatic cells to a totipotent state, undoing the epigenetic state of the somatic cell. In such experiments, we can observe that somatic cells lack the full epigenetic information that the germline has. It has sometimes been observed that cloned animals display a different epigenetic state from those of animals produced by germline: from the length of the teleomer to modified expression levels of genes [32]. The process that humans are trying to recreate is tightly related to the mechanism described in our paper. For a somatic cell to return to be a viable germline, epigenetic information that is lost during differentiation has to be recreated. Similarly, to create a second germline through evolution, organisms would evolve this developmental pathway. An interesting question is the source of this epigenetic information in the germline. Is all of it of genetic origin? How much information was generated epigenetically through epimutations? Our paper hints at the possibility that recreating the full state of the germline could be a hard task, if it includes epigenetic information that has been gathered over many generations, such that no developmental programme ever existed to generate the epigenetic state. The missing information could manifest in a single generation in the organism, but could also have subtle effects only manifest after many generations.

Finally, we note that the conflict between modes of information presented in this paper could occur in many major evolutionary transitions. A major transition results in the formation of new kinds of evolutionary individuals from pre-existing individuals—a higher-level individual composed of lower-level units [33]. The new individual must have some information that is common to its components. This information would also identify the lower-level units as belonging to the same higher-level unit. Yet to take advantage of being composed of lower-level units, i.e. specialization/modularity, there is likely to be information that is common to only a fraction of lower-level units (this information would be equivalent to the epigenetic markers discussed in this paper). In such cases, there would be a conflict concerning how these two modes of information are transferred to future generations. One resolution is evolution of a germline that safeguards the information of the higher-level unit.
